# Advancing Health Information Exchange: AI/ML, Social Needs, and Cybersecurity Trends

**DOI:** 10.1055/s-0046-1816622

**Published:** 2026-11-03

**Authors:** Joshua R. Vest, Haleigh Kampman, Ofir Ben-Assuli

**Affiliations:** 1 Indiana University Richard M Fairbanks School of Public Health, Department of Health Policy & Management, Regenstrief Institute, Center for Biomedical Informatics, Indianapolis, INUSA; 2 Indiana University Richard M Fairbanks School of Public Health, Department of Health Policy & Management, Eskenazi Health, Regenstrief Center for Health Equity Research, Indianapolis, INUSA; 3 Faculty of Business Administration, Ono Academic College, 104 Zahal Street, Kiryat Ono 55000Israel

**Keywords:** Health Information Exchange, Computer Security, Social Needs, Machine Learning, Review

## Abstract

**Objective** : Artificial intelligence and machine learning (AI/ML), social needs data, and cybersecurity are shaping the clinical and business environments that utilize and engage with health information exchange (HIE).

**Methods** : We reviewed the English language literature indexed in PubMed and Web of Science between 01/2019 and 11/2024. Articles were identified using the “health information exchange” MeSH major topic and as a phrase within titles and abstracts in combination with titles and keywords of the three topic areas. All three authors reviewed all full text articles for inclusion and excluded those that did not address HIE or our three emergent topics. From the full text, we extracted relevant themes and insights about the application of HIE.

**Results** : Articles on the application and role of AI/ML in HIE (n=12) were focused on using HIE data in AI/ML modeling or in applying AI/ML to information exchange technology process. Articles on social needs (n=13) included the themes of using individual-level or area-level social needs data in conjunction with data from HIE for research, using social needs data for care delivery and public health activities, or on the quality and usability of social needs data. Cybersecurity articles (n=10) focused on the use of HIE in response to cybersecurity threats and HIE design against cybersecurity threats.

**Conclusions** : The application and importance of AI/ML, social needs, and cybersecurity issues helps illustrate how far HIE has progressed from innovation to functioning as a foundational infrastructure technology for healthcare organizations and systems.

## 1. Introduction

Research on health information exchange (HIE), the process of electronically sharing data between different organizations [ 1 ], is perpetually on shifting and emerging ground. Overall, health information technology (HIT) that supports clinical care and healthcare organizations' business operations are evolving at a rapid pace. These innovations extend to the actual technology used to facilitate the sharing of patient level data, as well as new methodologies and capabilities to extract value from those data for clinical care and organizational decision making. So too, health policy and clinical practice environments are continually changing, bringing new potential use cases, opportunities, challenges, and even requirements to put data to use. On the one hand, this creates excitement and relevancy for HIE research. In many countries, HIE is a fundamental requirement and objective of health care policy and care delivery activities [ 2 3 4 ] and the newest technological innovations have potential applications to HIE. On the other hand, both the rapid changes in the technology and policy environments present a challenge in understanding the evidence on important developments in HIE.

The literature already includes expansive systematic reviews of HIE that catalog and report on the complete trajectory of information exchange and research sharing with particular attention to usage and outcomes [ 5 6 7 8 9 ]. However, in addition to these reviews, the literature also includes many and varied focused reviews on different use cases, aspects, and applications of HIE. For example, existing reviews cover the usage of HIE in inpatient care [ 10 ], in primary care [ 11 ], in transitions to long term care [ 12 ], in emergency medical services [ 13 ], in accountable care organizations [ 14 ], and in international settings [ 2 15 16 17 ]. Additionally, individual literature reviews have chronicled patient privacy concerns [ 18 ] and patient's attitudes [ 19 ]. Yet more literature reviews have covered topics as diverse as: use of HIE as research databases [ 20 ], HIE as a strategy to reduce readmissions [ 21 ], HIE use in sharing imaging studies [ 22 ], HIE use in pandemics [ 23 ], HIE and physician leadership [ 24 ], application of blockchain to interoperability [ 25 ], and even how to define HIE [ 26 ]. The rapid changes in HIT and policy may partially explain the variety and number of such reviews on HIE. For example, the most expansive systematic reviews of HIE often reported on the technologies, exchange networks, policy requirements, or clinical workflows as reported in the older pioneering studies of HIE and HIT. While critically informative, the pioneering works on HIE may not be accurate reflections of the current clinical and technological environments. In fact, some reviews have specifically argued for only the inclusion of more recent studies for this reason [ 27 28 ]. Additionally, the pace of change may also explain why many of these focused reviews span relatively short periods of time and frequently synthesize the findings of only a dozen or more studies.

In similar fashion, this review attempts to inventory and synthesize key trends relevant to health care operations and HIE today. For this review, we selected three emerging topics: artificial intelligence and machine learning (AI/ML), social needs data, and cybersecurity. All three of these topics are shaping the clinical and business environments that engage in HIE and are also emerging topics for which HIE may be potentially utilized. First, AI/ML dominates trade publications' and consultants' lists of important health care recent trends [ 29 30 31 ]. Additionally, AI/ML offers promising methodologies to leverage “big” data and HIE enables healthcare organizations and health information organizations to access immense repositories of clinical information [ 32 ]. Second, health systems around the world have identified social needs as critical drivers of healthcare quality and equity [ 33 ]. To see improvement in these areas, healthcare organizations must collect data on patients' health-related social needs (HRSNs) and the context in which patients live and work, i.e., their social determinants of health (SDOH) [ 34 ], and use these data to coordinate services across organizations and sectors [ 35 ]. Access to and sharing of data are exactly the problem that HIE was designed to address [ 36 ]. Lastly, healthcare organizations' data and HIT infrastructures are facing a growing set of cybersecurity threats, actions and events that create vulnerabilities and expose technology systems and data resources to damage or loss [ 37 ]. Over the past decade, data breaches and ransomware attacks have increased [ 38 ], so have disasters that have upended access to electronic health records (EHRs) [ 39 ]. HIE has been identified as a source to support healthcare organizations and patients during such events [ 40 ].

## 2. Methods

For the purposes of this narrative review, eligible articles could describe HIE as: a) the technical process of sharing medical information; b) the description of an organizational network, i.e., a network utility, that facilitates information sharing; c) a repository that combines data from multiple different decentralized sources; or d) a combination of preceding perspectives [ 26 ].

### 2.1 Search Strategy

For this review, we followed parallel search strategies for each of the three topics. JV and HK lead all database searches ( Figure 1 ). To identify relevant articles on HIE and AI/ML, we searched PubMed using the “health information exchange” MeSH major topic and as a phrase within titles and abstracts along with “machine learning” OR “artificial intelligence” as title and abstract terms. Because natural language processing (NLP) falls under the category of AI/ML, we also included it as a title/abstract term. From Web of Science, we entered the same terms in a search of titles and abstracts. For cybersecurity, we repeated the same approach for the HIE terms and used the “computer security” MeSH major term along with “hack”, “breach”, “attack”, “disaster”, OR “cybersecurity” as title and abstract terms. Lastly, we repeated the HIE searches and with the title and abstract keywords for social needs, i.e., “social determinants of health”, “social risk”, OR “social need”. Because we followed a parallel strategy for each topic, it is possible that a single article addressed more than one of our topics.

**Figure 1.**
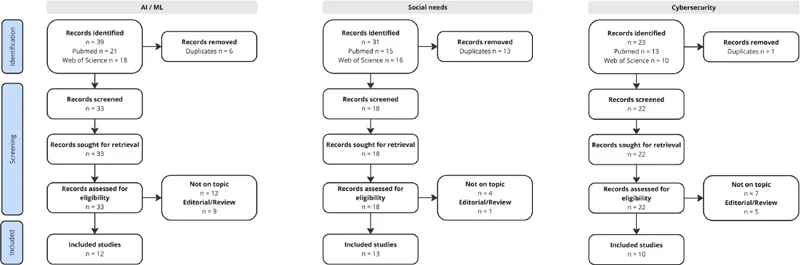
HIE literature survey - PRISMA FORMAT.

**Figure 2.**
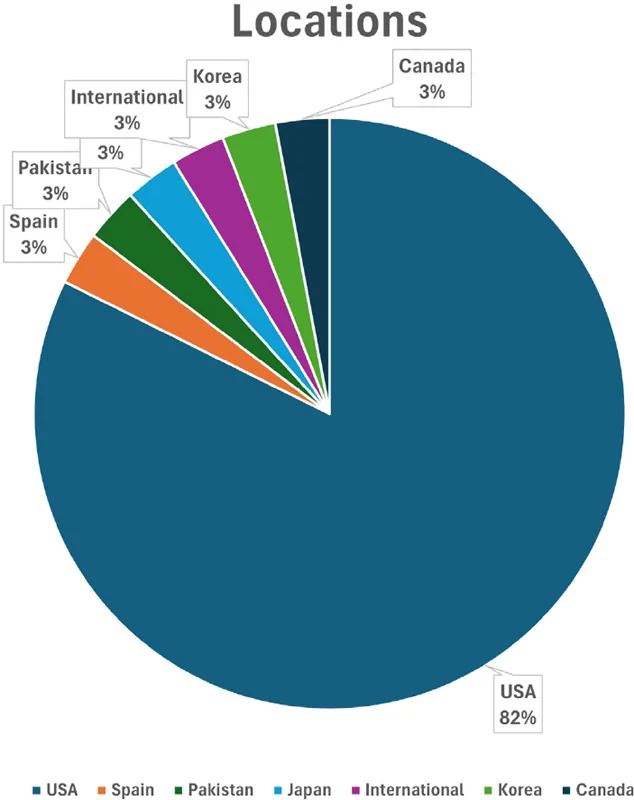
Articles by country of setting.

**Figure 3.**
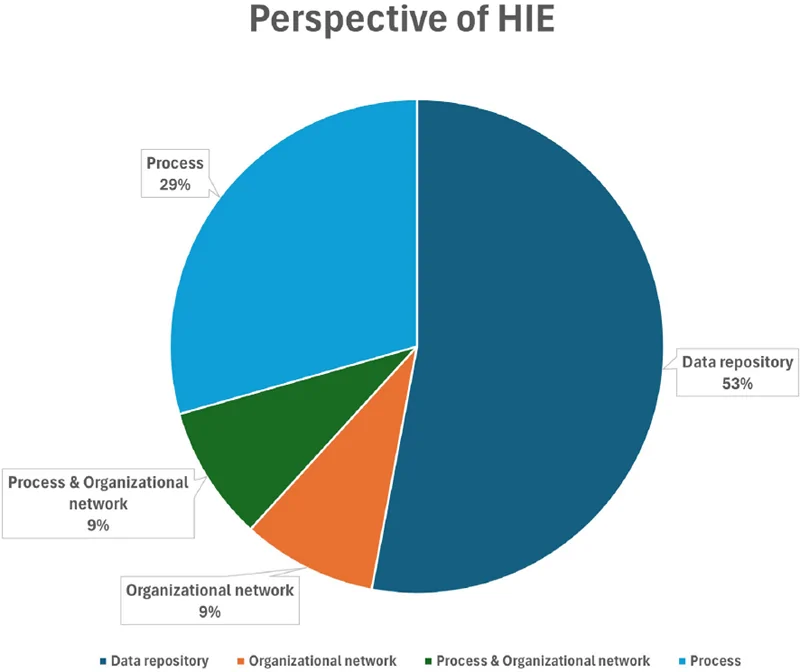
Articles by perspective of health information exchange.

### 2.2 Inclusion criteria

For all searches, results were limited to the past five years, which included articles published between January 2019 and November 2024. We selected this date as it covers the period after two major reviews on HIE [ 27 41 ]. Articles could discuss any clinical practice setting, use any study design, or describe HIE efforts in any country. However, to be included the article had to be published in English. Conference papers were eligible for inclusion.

### 2.3 Exclusion criteria

We excluded editorials and prior literature reviews. Our search strategy precluded pre-prints and gray literature. We did not include any exclusion criteria related to sample size or study quality (given the heterogeneous nature of the eligible literature, we did not assess study quality).

### 2.4 Assessment & Extraction

All three authors reviewed all full text articles for inclusion and excluded those that did not address HIE or our three emergent topics. Inclusion decisions were made by consensus; in instances of disagreement or uncertainty for inclusion, we jointly reviewed and discussed the articles to arrive at consensus. From parallel readings of the full text of articles from each topic area, HK and JV extracted relevant themes and insights about the application of HIE. To characterize the articles, HK and JV abstracted the location, setting (i.e., specific clinical setting or community-wide), study design, and the perspective from which the article discussed HIE. For authors from institutions in other countries, but who were using USA policies and technologies as the basis of discussion or context, their articles were listed as USA studies.

## 3. Results

The search strategy resulted in 73 unduplicated articles, of which 34 total articles meet our inclusion criteria ( Supplemental Table A ). Most of the studies identified from our search strategy were focused on HIE located in the USA (82%) ( Figure 2 ) The perspective from which the article approached HIE varied ( Figure 3 ). The majority considered, or used, HIE as a data repository for analyses (53%). This perspective was the most common for those dealing with AI/ML and social needs. The articles focused on cybersecurity universally approached HIE from the perspective of technical processes and/or as an organizational network. Few studies (15%) used longitudinal designs. Cross-sectional analyses (44%) and case studies (21%) were more common ( Supplemental Figure A ).

### 3.1 Artificial Intelligence and Machine Learning (AI/ML)

Our search strategy identified 12 relevant articles using our AI/ML and HIE search strategy. The most dominant theme within this set of studies was the application of ML techniques to various classification and prediction problems using data derived from HIE, e.g., using HIE as a data repository.

#### 3.1.1 Theme 1: Using HIE as a data repository for AI/ML modeling

Specifically, ML models were applied to: predicting whether laboratory reports were positive for different notifiable diseases [ 42 ], predicting future emergency department utilization [ 43 44 ], predicting the risk of heart failure for patients [ 45 ], identifying patients with a high risk of insomnia [ 46 ], predicting which patients will be in need of care for depression [ 47 ], identification of patients with hepatitis C lost to follow-up care [ 48 ], predicting future healthcare utilization among COVID-19 patients [ 49 ], and identifying peripheral arterial disease in clinical notes with deep learning [ 50 ]. With the exception of the last study, random forest [ 42 47 ], XGBoost [ 46 ], Hidden Markov models [ 43 ], and boosted-decision trees [ 44 45 ] were the selected ML models. These studies predominately relied on structured data elements only. The exceptions being the identification of positive laboratory reports [ 42 ], Kasthurirathne and colleagues' work on depression care [ 47 ], and the NLP work on peripheral arterial disease that used RoBERTa (Robustly Optimized Bidirectional Encoder Representations from Transformers Pretraining Approach) [ 50 ]. Notably, half of these studies leveraged an HIE data source from the same state [ 42 43 44 46 47 49 50 ].

In addition to demonstrating applicability to a wide set of clinical problems, this small set of studies provides broader insights into the use of HIE data for AI/ML modeling. One value noted was the advantage of existing data. For example, Duong and colleagues' [ 45 ] model predicting new onset heart failure using secondary HIE data had similar performance to risk scores that would require specific data collection. Additionally, our own work [ 44 ] suggests that HIE data may be more useful in prediction than data obtainable from a single institution. Third, use of HIE data would enable ML models to be deployed at scale and across multiple EHR systems [ 42 45 46 48 ]. As a potential limitations, few studies were attentive to the potential challenges of bias due to underrepresented populations in the data sources [ 46 49 ] or questionable data quality [ 45 ].

#### 3.1.2 Theme 2: Applying AI/ML to Information Exchange

A second, smaller theme within this set of articles was in applying AI/ML to the processes of exchange and HIE as an organizational network. As an example of this later type of study, McMoran and colleagues [ 51 ] used AI/ML to better understand the HIE network itself. Viewing the HIE as a network of EHRs and practices, this study used AI/ML techniques to describe patient distribution and provider participation. Three other articles offered more direct inclusion of AI/ML in HIE. Whereas other studies used secondary HIE data for prediction and classification models, Su and colleagues [ 52 ] described the application of classification and modeling as part of an HIE's architecture. Likewise, Zahoor and colleagues [ 53 ] describe an in-development information exchange framework for Pakistan that would incorporate real time data classification.

### 3.2 Social Needs

Our search strategy identified 13 relevant articles that discussed varying efforts using social needs (HRSNs and SDOH) data. We observed three general themes within this set: use of social needs data in conjunction with clinical data obtained from HIE sources for research; use of social needs as part of HIE activities directed at healthcare delivery and public health applications; and the quality and usability of social needs data sources for use in HIE.

#### 3.2.1 Theme 1: Use of Social Needs Data in Conjunction with HIE for Research

Most articles described combining social needs data elements with HIE clinical data within the context of a research question [ 44 54 55 56 57 58 59 60 ]. Within this set, these articles could be further subdivided into those that used social needs data to simply improve the accuracy of modeling various utilization outcomes [ 44 54 55 56 58 ] and those that used the combined data sources as a means to better understand potential disparities in patient reported outcomes [ 59 ] and hospital readmissions during the COVID-19 pandemic [ 60 ]. Of this set, the studies by Guo and colleagues [ 56 ] and Maguire and colleagues [ 57 ] are different in that they both considered SDOH (i.e., area-level social data) in conjunction with hospitals' adoption of HIE. Specifically, Guo and colleagues [ 56 ] found the associations between SDOH and emergency department utilization varied by hospitals' use of HIE. Using multiple measures of HIE adoption and usage, Maguire and colleagues [ 57 ] reported that hospitals located in geographical areas facing a greater burden of SDOH reported less information sharing capacity and activity. Also of note, most of these studies relied on aggregated area-level measures of SDOH from secondary data sources, such as zip codes or census-tract indicators [ 54 56 57 59 60 ]. Of the two studies that used patient-level HRSNs data, Gingold and colleagues [ 55 ] derived measures from existing EHR data (e.g., employment status, housing, and social support), but noted challenges with these data due to inconsistent documentation. Likewise, Mazurenko and colleagues [ 58 ] also used HRSNs extracted from clinical repositories, as well as primary survey data on HRSNs, and area-level SDOH measures.

#### 3.2.2 Theme 2: Use of Social Needs Data for Care Delivery & Public Health

As opposed to the first theme's focus on research use, a second theme revolved around application of social needs in care delivery and public health contexts [ 61 ]. For example, Richwine and colleagues [ 61 ] used the American Hospital Association (AHA) Annual Survey data to examine the landscape of hospitals' collection of social needs data. Per their analyses, most hospitals reported collecting some type of HRSN (i.e., patient-level social needs) data. The linkage to HIE was that those collecting HRSNs, about three-fourths reported using the information for referrals and coordination with external partner organizations. Beyond adoption by a single healthcare delivery organization, two articles described the development or enhancement of HIE systems with social needs data. A team in Texas [ 62 ] developed and piloted a shared information platform that linked patient-level HRSN screening and referral data with clinical information from the local HIE organization across two Federally Qualified Health Centers (FQHCs). The goal of both projects was to use shared information to improve coordination in a patient's follow-up to social service referrals. In Michigan, a team took a broader view of HIE systems – in effect, considering it just one of multiple sources of information that can be combined for population health [ 63 ]. The team described a state-level platform which would provide HIE and social needs data at the patient-level detail and aggregated data for multiple stakeholders to strengthen public health surveillance, improve care coordination, and support health policy. While the visions and activities of the respective teams in Texas and Michigan differ, both articles described the importance of having a partner with the existing exchange infrastructure, data management processes, security, and identity matching to support such efforts (all of these similarities are main HIE operational elements). Likewise, both noted that access to all the desired data to achieve their respective goals was a challenge.

#### 3.2.3 Theme 3: Quality and Usability of Social Needs Data

The third theme included two papers that were concerned with assessing and ensuring that social needs data were fit for use in HIE. For example, a national panel of HIE, EHR, and social care experts identified social needs-relevant data elements available in EHRs or via HIE and assessed the quality and potential biases of these elements [ 64 ]. The panel recommendations indicated that almost 90 data elements were relevant to patient's HRSNs, but that many could be subject to implicit biases or biases inherent in the healthcare system. Lastly, facing the increase in HRSN screening and the wide variety of different screening tools, Holt and colleagues [ 65 ] reported on an effort to map 15 different HRSN screeners to the standardized Simplified Omaha System Terms (SOST) taxonomy. They successfully mapped 98% of the screening items to SOST, which authors reported as an opportunity to facilitate more widespread exchange of patient's health-related social needs information.

### 3.3 Cybersecurity

Our search strategy identified 10 relevant articles in cybersecurity and HIE. The literature may be divided into two general themes.

#### 3.3.1 Theme 1: Use of HIE in Response to Cybersecurity Threats

The smaller theme was the role of HIE in situations that disrupted normal health care information technology and organizational operations due to disasters or data breaches. For example, in response to the need for shared information from different health systems during the COVID-19 pandemic, a team deployed a version of the OpenMRS 1 platform, an open source medical record system to collect basic clinical information and to replicate limited EHR functions [ 66 ]. The demonstration leveraged the master patient index and existing information from the statewide HIE. Likewise, in response to the 2011 Great East Japan Earthquake, a team designed and deployed an information exchange to support renal failure patients [ 67 ]. The other article within this theme took the alternative approach. Instead of using HIE as a means of mitigating disasters, the authors asked if hospital engagement with HIE increased the risk of a data breach [ 68 ]. Using survey and publicly reported data, the authors reported that breaches were rare, but that USA hospitals were more likely to have a breach of health information in the third year after HIE adoption.

#### 3.3.2 Theme 2: HIE Design Against Cybersecurity Threats

The remainder of the articles in this set could be described as technical and design issues. Case studies and simulation studies suggested blockchain [ 69 70 71 ], the privacy afforded by non-fungible patient tokenization [ 69 ], or new exchange protocol [ 72 ] as technical solutions to improve HIE cybersecurity while still remaining attentive to the needs of accessible and usable data [ 70 71 ]. These more technical papers were complimented by several qualitative studies. For example, qualitative work in the USA with academic physicians found general agreement that blockchain could support secure HIE [ 73 ]. Similarly, qualitative research in the Republic of Korea found that physicians and information technology professionals were aware of blockchain, whereas patients were not [ 74 ]. This same study reported that physicians and information technology professionals were more concerned about security than patients. Lastly, a qualitative study in Canada reported that patients did not believe concerns about security breaches should stop information exchange [ 75 ].

## 4. Discussion

All technologies have some trajectory, or path by which they develop and change over time, from being a novel innovation to developing into infrastructure, i.e., routine and foundational components required for daily operations and activities [ 76 77 ]. This review paper of AI/ML, social needs, and cybersecurity issues helps illustrate how HIE has progressed from an innovation to now functioning as a foundational infrastructure technology. The trajectory by which any technology progresses towards foundational infrastructure is the result of the combined demands of the environment, user needs, and other developments among related technologies [ 76 ]. In the case of HIE, the earliest efforts in the USA and Europe began as a series of local experiments [ 78 79 80 81 ]. Nevertheless, ambitions were greater. In the USA, the reports of the National Committee on Vital & Health Statistics were among the earliest calls for a nationwide health information infrastructure, which included information exchange as a component. Other nations similarly envisioned HIE as national technology that could support clinical care quality, costs, and public health [ 17 ]. Since that time, internet connectivity has improved, processing power and storage capacity have increased, and international standards have matured. Today, HIE is less an innovation and more a critical data repository and an organizational network with sets of processes to be optimized to reach national and international health goals. That role of HIE as foundational technology infrastructure is clearly articulated in the objectives set forth in recent policies, such as the European Health Data Space for Europe [ 82 ] and the Trusted Exchange Framework and Common Agreement (TEFCA) in the USA [ 83 ].

First, as demonstrated by the large number of studies included in this review [ 42 43 44 45 46 47 48 49 50 54 55 56 58 59 60 61 64 65 ], due to their immense amount of longitudinal and multi-institutional data, HIE is foundational to numerous research activities. This was the case for both the social needs and the AI/ML sets of studies. Studies leveraging HIE data overcome the limitations of single institution studies, are truly population-level type analyses, and can explore a wider array of outcomes. The research potential of HIE data repositories has long been recognized as a valuable use case [ 84 ]. This value remains, but the growing availability of de-identified claims databases and de-identified extracts of multiple health systems' EHRs from commercial sources is a serious challenge to the value of data derived from HIE. Nevertheless, HIE data repositories retain one potential strategic advantage, data in HIE repositories can be readily connected back to the care delivery processes. In particular, HIE networks are potentially well positioned to offer analytic services and additional off-EHR decision support through standards like Clinical Decision Support (CDS) Hooks [ 85 ], Substitutable Medical Applications, Reusable Technologies (SMART) [ 86 ], and Fast Healthcare Interoperability Resources (FHIR) [ 87 ]. Thus, innovations have a natural setting for movement to implementation and testing in real-world environments. Future work that draws on HIE as a data repository would help support the utility and sustainability of HIE by translating findings into interventions. Likewise, through connections to all types of settings, HIE represents an opportunity for increased dissemination of more advanced analytics and decision support at the point of care. Necessary to forwarding such usage is an attentiveness to fair and unbiased performance.

Additionally in the social needs set of literature, HIE organizational networks demonstrate progression to foundational infrastructure through the ability to extend healthcare delivery into new domains and sectors of the economy to support broader gains in health [ 62 63 ]. These examples are only feasible because of the opportunity to build upon existing technological investments that enable the linking and secure sharing of patient records. In the USA, which does not have a true national unique patient identifier, this foundational technology extends to identity management. As more and more community-based and social care organizations become key players in the healthcare system, it is arguable that health information organization's community-wide master patient indexes may be among their more valuable resources allowing for closed-loop referrals and patient follow-up across services. In addition, HIE networks are effectively interorganizational information networks and one means to increase the value of such networks is to increase the number of participants [ 88 ]. Given the increasing importance of HRSNs, social service and community-based organizations are an obvious target for HIE expansion. Problematically, many potential partners in the social service sector are both low-resourced and less technologically sophisticated. As such, this sector is likely not a source of revenue. Instead, the primary value in adding community-based organizations to an HIE network will be in increasing healthcare organizations' access to new and novel data on social needs and partners for service referrals. However, as the example in this review noted, to leverage any new and existing data will require continued progression on, and adoption of, emerging international data standards for HRSN and SDOH measurements and observations [ 89 ]. Standards would also support research and analytics opportunities as well.

While not appearing extensively in this review, larger, more diverse networks, with standard data open the opportunities for better use of deep learning methods with HIE systems and its related data. To be ready for more powerful analytic models for use in individual care and population health, operators of HIE systems and researchers need to be better positioned to deploy explainable artificial intelligence (xAI) tools. xAI tools seek to increase transparency and understandability of complex AI/ML models [ 90 ], but these tools themselves may be quite complex [ 91 ]. While integration of xAI tools into decision support systems, for example, has advantages to end user understanding above and beyond providing accuracy measures, the integration of xAI requires thoughtful and intentional integration [ 92 ]. In a similar vein, visual analytics could also prove useful in turning the longitudinal data available from HIE systems into information or decision-making [ 93 94 ]. Application of such tools that enable end users to better understand the potential shortcoming and challenges associated with analytic models can potentially help mitigate, or at least account for, biases in model performance and the underlying data. Such tools help keep end users closer to the processes that result in model conclusions. Regardless of the methodological advances applied in this area, future research needs to remain attentive to the possibility of inequitable performance of AI/ML models [ 95 ]. While HIE data avoid the potential problems of a single institutional source, all real-world healthcare data are reflections of the differential access opportunities and experiences inherent in systems of care delivery. Ensuring fairness and addressing bias should be parts of all analyses and clearly reported [ 96 97 ].

HIE is also a foundational infrastructure for the healthcare system given its potential application in response to disasters or cyber-attacks. Thinking in this context of HIE as a noun has key characteristics. Most notably, HIE networks function as a source of data redundancy for any single participating healthcare organization and, by extension, their patients. For example, patient data remains intact and accessible even if physical care locations are damaged, rendered inaccessible, or destroyed. The same is true for healthcare organizations that fall prey to ransomware or hacking events. During such events, clinicians and providers face a significant need for information [ 98 ]. Health professionals can access and review patient records electronically, even from temporary facilities. Examples of this use have occurred after flooding [ 99 ] and tornados [ 100 ] and this use case was envisioned by articles included in this review [ 66 67 ]. As the risk to healthcare organizations from cyberattacks and costly natural disasters increases, a redundant source of patient information could be a need or business opportunity for health information organizations offering HIE services to fill for the industry. However, while access to information is necessary during such events, it is not a complete replacement for the key functionality offered by an EHR. Clinicians and organizations also need a means of electronic documentation and orders [ 98 ]. Access to information was the primary goal of the HIE system to support renal failure patients [ 67 ] and, to a lesser extent, of the work in Indiana during COVID-19, as this team also focused on functionalities like patient registration [ 42 45 46 48 66 ]. Health information organizations seeking to fulfill this role of redundant systems will need to build upon such works and offer additional functionality that supports core business and operating functions in addition to those necessary to deliver clinical care. Likewise, as the articles included in this review pointed out, security of HIE remains paramount and will have to continue to evolve to avoid and respond to the increasingly difficult environment. Further research is needed on the effects of EHR outages on HIE networks.

### 4.1 Limitations

This review is limited in scope to a select set of emerging topics and by a narrow date range. As already suggested in this paper, work on HIE is being disseminated rapidly and we may have missed some recently published studies by our exclusion of pre-prints and the gray literature. While our search strategy included databases that included sources beyond the healthcare literature, inclusion of other sources may have identified other relevant articles. Similarly, we did not conduct any hand searching or include non-English language sources and doing so, with proper interpretation, may have broaden our findings. Likewise, focus on English language may have biased our findings towards research based in the USA. In addition, our three themes (AI/ML, social needs, and cybersecurity) are themselves broad and highly variable topics. Individual reviews on any of these topics would be better positioned to explore the nuances and variations in methods and definitions. Lastly, we did not assess the articles for potential bias or overall quality. Therefore, publication bias may have excluded more critical studies or null findings. Most studies were descriptive in nature or single site case studies, which limits generalizability.

### 4.2 Avenues for Future Research

The literature included in this review and the above discussion suggest several avenues for potential HIE research. First, AI/ML and cybersecurity cannot be considered completely independently. While AI/ML may be used to support cybersecurity, the resources, partners, and extent of data needed and used in AI/ML applications may increase risk to privacy and data breaches [ 101 ]. Second, as noted in the reviewed studies, social data and health care data are susceptible to biases [ 58 64 ], but efforts at integrating these two sources are ongoing [ 62 63 ]. Unfortunately, AI/ML can often amplify such biases. Establishing whether access to more information sources via HIE can mitigate biased data or if the shared repositories made possible by HIE could support new analytic approaches and bias mitigation strategies is an area for future work at the intersection of AI/ML and social needs. Lastly, if, as we contend, HIE is moving towards a foundational technology, an additional area for future research will be identifying the tactics to extract value from HIE as an organizational and public resource. This moves beyond potential cost savings from using HIE, which have been previously identified and catalogued [ 7 102 ]. Instead, we suggest the need for quantification of the value of HIE to organizational strategic goals and activities for health care organizations and for public policy. Demonstrating that HIE (whether conceptualized as a technical process of information sharing, participation in an exchange network, or a data repository) is necessary to organizational and policy goals would be one path to justifying the use case for sustainability.

## 5. Conclusion

Healthcare and health policy continue to change and evolve and require technology to support those changes. HIE data and technologies play a key role in healthcare's increasing use of AI/ML techniques. HIE is positioned to support healthcare's increasing focus on social needs and determinants. Lastly, HIE can support organizations during disruptions to normal organizational operations, as well as be a place to innovate in security approaches. HIE has progressed from innovation to functioning as a foundational infrastructure technology for healthcare.

## Footnotes

1
https://openmrs.org/

## References

[1] The National Alliance for Health Information Technology. Report to the Office of the National Coordinator for Health Information Technology on Defining Key Health Information Technology Terms [Internet]. Washington, DC: US Department of Health & Human Services; 2008. Available from:http://healthit.hhs.gov/portal/server.pt?open=18&objID=848133&parentname=CommunityPage&parentid=5&mode=2&in_hi_userid=10741&cached=true

[2] Mamuye AL, Yilma TM, Abdulwahab A, Broomhead S, Zondo P, Kyeng M, *et al.* Health information exchange policy and standards for digital health systems in africa: A systematic review. PLOS Digit Health. 2022;1(10):e0000118. https://doi.org/10.1371/journal.pdig.0000118 10.1371/journal.pdig.0000118 PMC9931258 36812615

[3] Payne TH, Lovis C, Gutteridge C, Pagliari C, Natarajan S, Yong C, *et al.* Status of health information exchange: a comparison of six countries. J Glob Health . 2019;9(2):020427. https://doi.org/10.7189/jogh.09.020427 10.7189/jogh.09.020427 PMC6815656 31673351

[4] Holmgren AJ, Esdar M, Hüsers J, Coutinho-Almeida J. Health Information Exchange: Understanding the Policy Landscape and Future of Data Interoperability. Yearb Med Inform. 2023;32(1):184–94.https://doi.org/10.1055/s-0043-1768719 10.1055/s-0043-1768719 PMC10751121 37414031

[5] Devine EB, Totten AM, Gorman P, Eden KB, Kassakian S, Woods S, *et al.* Health Information Exchange Use (1990-2015): A Systematic Review. EGEMS (Wash DC). 2017;5(1):27. https://doi.org/10.5334/egems.249 10.5334/egems.249 PMC5983051 29881743

[6] Eden KB, Totten AM, Kassakian SZ, Gorman PN, McDonagh MS, Devine B, *et al.* Barriers and facilitators to exchanging health information: a systematic review. Int J Med Inform. 2016;88:44–51. https://doi.org/10.1016/j.ijmedinf.2016.01.004 10.1016/j.ijmedinf.2016.01.004 PMC4778080 26878761

[7] Hersh WR, Totten AM, Eden KB, Devine B, Gorman P, Kassakian SZ, *et al.* Outcomes From Health Information Exchange: Systematic Review and Future Research Needs. JMIR Med Inform. 2015;3(4):e39. https://doi.org/10.2196/medinform.5215 10.2196/medinform.5215 PMC4704923 26678413

[8] Rahurkar S, Vest JR, Menachemi N. Despite the spread of health information exchange, there is little evidence of its impact on cost, use, and quality of care. Health Aff (Millwood). 2015;34(3):477–83.https://doi.org/10.1377/hlthaff.2014.0729 10.1377/hlthaff.2014.0729 25732499

[9] Rudin RS, Motala A, Goldzweig CL, Shekelle PG. Usage and effect of health information exchange: a systematic review. Ann Intern Med. 2014;161(11):803–11.https://doi.org/10.7326/M14-0877 10.7326/M14-0877 25437408

[10] Dupont S, Nemeth J, Turbow S. Effects of Health Information Exchanges in the Adult Inpatient Setting: a Systematic Review. J Gen Intern Med. 2023;38(4):1046–53.https://doi.org/10.1007/s11606-022-07872-z 10.1007/s11606-022-07872-z PMC10039134 36376635

[11] Fontaine P, Ross SE, Zink T, Schilling LM. Systematic review of health information exchange in primary care practices. J Am Board Fam Med. 2010;23(5):655–70.https://doi.org/10.3122/jabfm.2010.05.090192 10.3122/jabfm.2010.05.090192 20823361

[12] Kruse CS, Marquez G, Nelson D, Palomares O. The Use of Health Information Exchange to Augment Patient Handoff in Long-Term Care: A Systematic Review. Appl Clin Inform. 2018;9(4):752–71.https://doi.org/10.1055/s-0038-1670651 10.1055/s-0038-1670651 PMC6170191 30282094

[13] Martin TJ, Ranney ML, Dorroh J, Asselin N, Sarkar IN. Health Information Exchange in Emergency Medical Services. Appl Clin Inform. 2018;9(4):884–91.https://doi.org/10.1055/s-0038-1676041 10.1055/s-0038-1676041 PMC6291398 30541153

[14] Balio CP, Apathy NC, Danek RL. Health Information Technology and Accountable Care Organizations: A Systematic Review and Future Directions. EGEMS (Wash DC). 2019;7(1):24.https://doi.org/10.5334/egems.261 10.5334/egems.261 PMC6625537 31328131

[15] Akhlaq A, McKinstry B, Muhammad KB, Sheikh A. Barriers and facilitators to health information exchange in low- and middle-income country settings: a systematic review. Health Policy Plan. 2016;31(9):1310–25.https://doi.org/10.1093/heapol/czw056 10.1093/heapol/czw056 27185528

[16] Li E, Clarke J, Ashrafian H, Darzi A, Neves AL. The Impact of Electronic Health Record Interoperability on Safety and Quality of Care in High-Income Countries: Systematic Review. J Med Internet Res. 2022;24(9):e38144.https://doi.org/10.2196/38144 10.2196/38144 PMC9523524 36107486

[17] Vest JR. Health information exchange: national and international approaches. Adv Health Care Manag. 2012;12:3–24.https://doi.org/10.1108/s1474-8231(2012)0000012005 10.1108/s1474-8231(2012)0000012005 22894043

[18] Shen N, Bernier T, Sequeira L, Strauss J, Silver MP, Carter-Langford A, *et al.* Understanding the patient privacy perspective on health information exchange: A systematic review. Int J Med Inform. 2019;125:1–12. https://doi.org/10.1016/j.ijmedinf.2019.01.014 10.1016/j.ijmedinf.2019.01.014 30914173

[19] Esmaeilzadeh P, Sambasivan M. Patients' support for health information exchange: a literature review and classification of key factors. BMC Med Inform Decis Mak. 2017;17(1):33.https://doi.org/10.1186/s12911-017-0436-2 10.1186/s12911-017-0436-2 PMC5379518 28376785

[20] Garza M, Myneni S, Nordo A, Eisenstein EL, Hammond WE, Walden A, *et al.* eSource for Standardized Health Information Exchange in Clinical Research: A Systematic Review. Stud Health Technol Inform. 2019;257:115–24. 30741183

[21] Kash BA, Baek J, Davis E, Champagne-Langabeer T, Langabeer JR. Review of successful hospital readmission reduction strategies and the role of health information exchange. Int J Med Inform. 2017;104:97–104.https://doi.org/10.1016/j.ijmedinf.2017.05.012 10.1016/j.ijmedinf.2017.05.012 28599821

[22] Vest JR, Jung HY, Ostrovsky A, Das LT, McGinty GB. Image Sharing Technologies and Reduction of Imaging Utilization: A Systematic Review and Meta-analysis. J Am Coll Radiol. 2015;12(12 Pt B):1371-1379.e3.https://doi.org/10.1016/j.jacr.2015.09.014 10.1016/j.jacr.2015.09.014 PMC4730956 26614882

[23] Massoudi BL, Sobolevskaia D. Keep Moving Forward: Health Informatics and Information Management beyond the COVID-19 Pandemic. Yearb Med Inform. 2021;30(1):75–83.https://doi.org/10.1055/s-0041-1726499 10.1055/s-0041-1726499 PMC8416200 34479380

[24] Heath ML, Porter TH. Physician leadership and health information exchange: literature review. BMJ Health Care Inform. 2019;26(1).https://doi.org/10.1136/bmjhci-2019-100080 10.1136/bmjhci-2019-100080 PMC7062346 31444230

[25] Schmeelk S, Kanabar M, Peterson K, Pathak J. Electronic health records and blockchain interoperability requirements: a scoping review. JAMIA Open. 2022;5(3):ooac068.https://doi.org/10.1093/jamiaopen/ooac068 10.1093/jamiaopen/ooac068 PMC9329659 35911668

[26] Akhlaq A, Sheikh A, Pagliari C. Defining Health Information Exchange: Scoping Review of Published Definitions. J Innov Health Inform. 2017;23(4):838.https://doi.org/10.14236/jhi.v23i4.838 10.14236/jhi.v23i4.838 28346130

[27] Menachemi N, Rahurkar S, Harle CA, Vest JR. The benefits of health information exchange: an updated systematic review. J Am Med Inform Assoc. 2018;25(9):1259–65.https://doi.org/10.1093/jamia/ocy035 10.1093/jamia/ocy035 PMC7646861 29718258

[28] Sarkar IN. Transforming Health Data to Actionable Information: Recent Progress and Future Opportunities in Health Information Exchange. Yearb Med Inform. 2022;31(1):203–14.https://doi.org/10.1055/s-0042-1742519 10.1055/s-0042-1742519 PMC9719753 36463879

[29] Marr B. Forbes. 2023 [cited 2024]. The 10 Biggest Trends Revolutionizing Healthcare In 2024. Available from:https://www.forbes.com/sites/bernardmarr/2023/10/03/the-10-biggest-trends-revolutionizing-healthcare-in-2024/

[30] Patel N, Singhal S. McKinsey & Company. 2024 [cited 2024]. What to expect in US healthcare in 2024 and beyond. Available from:https://www.mckinsey.com/industries/healthcare/our-insights/what-to-expect-in-us-healthcare-in-2024-and-beyond

[31] Gamble S, Gamble M. Becker's Hospital Review. 2024. 12 healthcare trends and issues we are following for 2024. Available from:https://www.beckershospitalreview.com/strategy/12-healthcare-trends-and-issues-we-are-following-for-2024.html

[32] Borna S, Maniaci MJ, Haider CR, Maita KC, Torres-Guzman RA, Avila FR, *et al.* Artificial Intelligence Models in Health Information Exchange: A Systematic Review of Clinical Implications. Healthcare. 2023;11(18):2584. https://doi.org/10.3390/healthcare11182584 10.3390/healthcare11182584 PMC10531020 37761781

[33] Donkin A, Goldblatt P, Allen J, Nathanson V, Marmot M. Global action on the social determinants of health. BMJ Glob Health [Internet]. 2018 ;3(Suppl 1).https://doi.org/10.1136/bmjgh-2017-000603 10.1136/bmjgh-2017-000603 PMC5759713 29379648

[34] Department of Health & Human Services. Medicare Program; Hospital Inpatient Prospective Payment Systems for Acute Care Hospitals and the Long-Term Care Hospital Prospective Payment System and Policy Changes and Fiscal Year 2023 Rates; Quality Programs and Medicare Promoting Interoperability Program Requirements for Eligible Hospitals and Critical Access Hospitals; Costs Incurred for Qualified and Non-Qualified Deferred Compensation Plans; and Changes to Hospital and Critical Access Hospital Conditions of Participation. Federal Register. 2022;87 (153):48780–9499. Available from:https://www.federalregister.gov/documents/2022/12/13/2022-26986/medicare-program-hospital-inpatient-prospective-payment-systems-for-acute-care-hospitals-and-the

[35] National Academies of Sciences Engineering, Medicine. Integrating Social Care into the Delivery of Health Care: Moving Upstream to Improve the Nation's Health [Internet]. Washington, DC: The National Academies Press; 2019. Available from:https://nap.nationalacademies.org/catalog/25467/integrating-social-care-into-the-delivery-of-health-care-movinghttps://doi.org/10.17226/25467 31940159

[36] Vest JR, Gamm LD. Health information exchange: persistent challenges and new strategies. J Am Med Inform Assoc. 2010;17 (3):288–94.https://doi.org/10.1136/jamia.2010.003673 10.1136/jamia.2010.003673 PMC2995716 20442146

[37] Jang-Jaccard J, Nepal S. A survey of emerging threats in cybersecurity. JCSS. 2014;80(5):973–93.https://doi.org/10.1016/j.jcss.2014.02.005

[38] Lee I. An analysis of data breaches in the U.S. healthcare industry: diversity, trends, and risk profiling. Inf. Secur. J.: Global Perspect. 2022;31(3):346–58.https://doi.org/10.1080/19393555.2021.2017522

[39] Horahan K, Morchel H, Raheem M, Stevens L. Electronic health records access during a disaster. Online J Public Health Inform. 2014;5(3):232.https://doi.org/2013 10.5210/ojphi.v5i3.4826 PMC3959913 24683443

[40] Shapiro JS, Mostashari F, Hripcsak G, Soulakis N, Kuperman G. Using Health Information Exchange to Improve Public Health. Am J Public Health. 2011;101(4):616–23.https://doi.org/10.2105/AJPH.2008.158980 10.2105/AJPH.2008.158980 PMC3052326 21330598

[41] Sadoughi F, Nasiri S, Ahmadi H. The impact of health information exchange on healthcare quality and cost-effectiveness: A systematic literature review. Comput Methods Programs Biomed. 2018;161:209–32.https://doi.org/10.1016/j.cmpb.2018.04.023 10.1016/j.cmpb.2018.04.023 29852963

[42] Dexter GP, Grannis SJ, Dixon BE, Kasthurirathne SN. Generalization of Machine Learning Approaches to Identify Notifiable Conditions from a Statewide Health Information Exchange. AMIA Jt Summits Transl Sci Proc. 2020;2020:152. PMC7233074 32477634

[43] Ben-Assuli O, Vest JR. Data mining techniques utilizing latent class models to evaluate emergency department revisits. J Biomed Inform. 2020;101:103341.https://doi.org/10.1016/j.jbi.2019.103341 10.1016/j.jbi.2019.103341 31747623

[44] Vest JR, Ben-Assuli O. Prediction of emergency department revisits using area-level social determinants of health measures and health information exchange information. Int J Med Inform. 2019;129:205–10.https://doi.org/10.1016/j.ijmedinf.2019.06.013 10.1016/j.ijmedinf.2019.06.013 31445257

[45] Duong SQ, Zheng L, Xia M, Jin B, Liu M, Li Z, *et al.* Identification of patients at risk of new onset heart failure: Utilizing a large statewide health information exchange to train and validate a risk prediction model. PLoS One. 2021;16 (12):e0260885. https://doi.org/10.1371/journal.pone.0260885 10.1371/journal.pone.0260885 PMC8664210 34890438

[46] Holler E, Chekani F, Ai J, Meng W, Khandker RK, Ben Miled Z, *et al.* Development and Temporal Validation of an Electronic Medical Record-Based Insomnia Prediction Model Using Data from a Statewide Health Information Exchange. J Clin Med. 2023;12(9). https://doi.org/10.3390/jcm12093286 10.3390/jcm12093286 PMC10179562 37176726

[47] Kasthurirathne S, Biondich P, Grannis S, Purkayastha S, Vest JR, Jones J. Identification of Patients in Need of Advanced Care for Depression Using Data Extracted From a Statewide Health Information Exchange: A Machine Learning Approach. Journal of Medical Internet Research. 2019;21(7).https://doi.org/10.2196/13809 10.2196/13809 PMC6681643 31333196

[48] Parada Vázquez P, Pérez-Cachafeiro S, Castiñeira Domínguez B, González-Pérez JM, Mera Calviño JM, Souto-Rodríguez R, *et al.* Artificial intelligence-assisted identification and retrieval of chronic hepatitis C patients lost to follow-up in the health area of Pontevedra and O Salnés (Spain). Gastroenterol Hepatol. 2024;502226. https://doi.org/10.1016/j.gastrohep.2024.502226 10.1016/j.gastrohep.2024.502226 38950646

[49] Kasturi SN, Park J, Wild D, Khan B, Haggstrom DA, Grannis S. Predicting COVID-19-Related Health Care Resource Utilization Across a Statewide Patient Population: Model Development Study. J Med Internet Res. 2021;23(11):e31337.https://doi.org/10.2196/31337 10.2196/31337 PMC8594735 34581671

[50] Dev S, Zolensky A, Aridi H, Kelty C, Madison M, Motaganahalli A, *et al.* Use of Deep Learning to Identify Peripheral Arterial Disease Cases From Narrative Clinical Notes. J Surg Res. 2024;303:699–708. https://doi.org/10.1016/j.jss.2024.09.062 10.1016/j.jss.2024.09.062 39454287

[51] McMoran D, O'Malley A, Karamustafaoglu A, Fisher D, Dogan G. Using Artificial Intelligence to Predict Patient Electronic Health Record Access Points. In: Wani M, Kantardzic M, Palade V, Neagu D, Yang L, Chan K, editors. 21st IEEE International Conference on Machine Learning and Applications (ICMLA). 2022. p. 969–73.https://doi.org/10.1109/ICMLA55696.2022.00162

[52] Su H, Yao L, Hou D, Sun M, Hou J, Ying J, *et al.* Cloud Computing Management Architecture for Digital Health Remote Patient Monitoring. In: 2021 IEEE International Conference on Smart Computing (SMARTCOMP). 2021. p. 209–14. https://doi.org/10.1109/SMARTCOMP52413.2021.00049

[53] Zahoor I, Ihtsham S, Ramzan M, Raza A, Ali B, Assoc Computing Machinery. AI-Driven Healthcare Delivery in Pakistan: A Framework for Systemic Improvement. In: Lahore University of Management Sciences. 2024. p. 30–7.https://doi.org/10.1145/3674829.3675058

[54] Ben-Assuli O, Vest JR. Return visits to the emergency department: An analysis using group based curve models. Health Informatics J. 2022;28(2).https://doi.org/10.1177/14604582221105444 10.1177/14604582221105444 35676746

[55] Gingold D, Liang Y, Stryckman B, Marcozzi D. The effect of a mobile integrated health program on health care cost and utilization. Health Serv Res. 2021;56(6):1146–55.https://doi.org/10.1111/1475-6773.13773 10.1111/1475-6773.13773 PMC8586470 34402056

[56] Guo T, Bardhan I, Khurshid A. Social Determinants of Health and ER Utilization: Role of Information Integration during COVID-19. ACM Trans. Manag. Inf. Syst. 2023;14(4).https://doi.org/10.1145/3583077

[57] Maguire T, Yoon S, Chen J. Collaborating for COVID-19: Hospital Health Information Exchange and Public Health Partnership. Telemed J E Health. 2024;30(1):108–17.https://doi.org/10.1089/tmj.2023.0056 10.1089/tmj.2023.0056 PMC10794829 37294562

[58] Mazurenko O, Hirsh AT, Harle CA, Shen J, McNamee C, Vest JR. Comparing the performance of screening surveys versus predictive models in identifying patients in need of health-related social need services in the emergency department. He Z, editor. PLoS ONE. 2024;19(11):e0312193.https://doi.org/10.1371/journal.pone.0312193 10.1371/journal.pone.0312193 PMC11578524 39565746

[59] Rana P, Brennan J, Johnson A, Turcotte J, Petre B. Social Determinants of Health in Maryland Hip Arthroscopy Patients. Cureus. 2024;16(1).https://doi.org/10.7759/cureus.52576 10.7759/cureus.52576 PMC10874623 38371015

[60] Sandoval M, Mikhail J, Fink M, Tortolero G, Cao T, Ramphul R, *et al.* Social determinants of health predict readmission following COVID-19 hospitalization: a health information exchange-based retrospective cohort study. Front Public Health. 2024;12. https://doi.org/10.3389/fpubh.2024.1352240 10.3389/fpubh.2024.1352240 PMC11004289 38601493

[61] Richwine C, Patel V, Everson J, Iott B. The role of routine and structured social needs data collection in improving care in US hospitals. J Am Med Inform Assoc. 2024;ocae279.https://doi.org/10.1093/jamia/ocae279 10.1093/jamia/ocae279 PMC11648711 39504474

[62] Khurshid A, Hautala M, Oliveira E, Lakshminarayanan V, Abrol V, Collier J, *et al.* Social and Health Information Platform: Piloting a Standards-Based, Digital Platform Linking Social Determinants of Health Data into Clinical Workflows for Community-Wide Use. Appl Clin Inform. 2023;14(05). https://doi.org/10.1055/s-0043-1774819 10.1055/s-0043-1774819 PMC10632068 37940130

[63] Korzeniewski SJ, Bezold C, Carbone JT, Danagoulian S, Foster B, Misra D, *et al.* The Population Health OutcomEs aNd Information Exchange (PHOENIX) Program - A Transformative Approach to Reduce the Burden of Chronic Disease. OJPHI [Internet]. 2020;12(1). Available from: https://journals.uic.edu/ojs/index.php/ojphi/article/view/10456 10.5210/ojphi.v12i1.10456 PMC7295585 32577152

[64] Vest JR, Adler-Milstein J, Gottlieb L, Bian J, Campion T, Cohen G, *et al.* Assessment of Structured Data Elements for Social Risk Factors. Am J Manag Care. 2022;28(1):E14–23. https://doi.org/10.37765/ajmc.2022.88816 10.37765/ajmc.2022.88816 PMC12968838 35049262

[65] Holt J, Austin R, Atadja R, Cole M, Noonan T, Monsen K. Comparison of SIREN social needs screening tools and Simplified Omaha System Terms: informing an informatics approach to social determinants of health assessments. J Am Med Inform Assoc. 2023;30(11):1811–7.https://doi.org/10.1093/jamia/ocad092 10.1093/jamia/ocad092 PMC10586032 37221701

[66] Mamlin B, Shivers J, Glober N, Dick J. OpenMRS as an emergency EMR-How we used a global good to create an emergency EMR in a week. Int J Med Inform. 2021;149.https://doi.org/10.1016/j.ijmedinf.2021.104433 10.1016/j.ijmedinf.2021.104433 PMC9760786 33752170

[67] Ido K, Miyazaki M, Nakayama M. Hemodialysis Record Sharing: Solution for Work Burden Reduction and Disaster Preparedness. JMIR Form Res. 2022;6(7).https://doi.org/10.2196/32925 10.2196/32925 PMC9356332 35867394

[68] Choi S, Chen M, Tan X. Assessing the impact of health information exchange on hospital data breach risk. Int J Med Inform. 2023;177.https://doi.org/10.1016/j.ijmedinf.2023.105149 10.1016/j.ijmedinf.2023.105149 37453177

[69] Zhuang Y, Shyu C, Hong S, Li P, Zhang L. Self-sovereign identity empowered non-fungible patient tokenization for health information exchange using blockchain technology. Comput Biol Med. 2023;157.https://doi.org/10.1016/j.compbiomed.2023.106778 10.1016/j.compbiomed.2023.106778 36934533

[70] Zhuang Y, Sheets LR, Chen YW, Shae ZY, Tsai JJP, Shyu CR. A Patient-Centric Health Information Exchange Framework Using Blockchain Technology. IEEE J Biomed Health Inform. 2020;24(8):2169–76.https://doi.org/10.1109/JBHI.2020.2993072 10.1109/JBHI.2020.2993072 32396110

[71] Zhuang Y, Zhang L. Promoting TEFCA with Blockchain Technology: A Decentralized Approach to Patient-centered Healthcare Data Management. AMIA Annu Symp Proc. 2023;2023:824–33. PMC10785864 38222410

[72] Mezher M, Hellmuth N, Ibrahim A, IEEE Comp Soc. Secure Health Information Exchange (S-HIE) Protocol with Reduced Round-Trip Count. In: Pennsylvania Commonwealth System of Higher Education (PCSHE). 2021. p. 280–8.https://doi.org/10.1109/ICHI52183.2021.00051

[73] Esmaeilzadeh P. Benefits and concerns associated with blockchain-based health information exchange (HIE): a qualitative study from physicians' perspectives. BMC Med Inform Decis Mak. 2022;22(1):80.https://doi.org/10.1186/s12911-022-01815-8 10.1186/s12911-022-01815-8 PMC8962111 35346176

[74] Lee K, Lim K, Jung SY, Ji H, Hong K, Hwang H, *et al.* Perspectives of Patients, Health Care Professionals, and Developers Toward Blockchain-Based Health Information Exchange: Qualitative Study. J Med Internet Res. 2020;22(11):e18582. https://doi.org/10.2196/18582 10.2196/18582 PMC7695529 33185553

[75] Shen N, Sequeira L, Silver M, Carter-Langford A, Strauss J, Wiljer D. Patient Privacy Perspectives on Health Information Exchange in a Mental Health Context: Qualitative Study. JMIR Ment Health. 2019;6 (11).https://doi.org/10.2196/13306 10.2196/13306 PMC6881785 31719029

[76] Dosi G. Technological paradigms and technological trajectories: A suggested interpretation of the determinants and directions of technical change. Research Policy. 1982;11(3):147–62.https://doi.org/10.1016/0048-7333(82)90016-6

[77] Rogers E. Diffusion of Innovations. 5th ed. New York: Free Press; 2003.

[78] Starr P. Smart Technology, Stunted Policy: Developing Health Information Networks. Health Aff. 1997;16(3):91–105.https://doi.org/10.1377/hlthaff.16.3.91 10.1377/hlthaff.16.3.91 9141326

[79] McDonald CJ, Overhage JM, Barnes M, Schadow G, Blevins L, Dexter PR, *et al.* The Indiana Network For Patient Care: A Working Local Health Information Infrastructure. Health Aff. 2005;24(5):1214–20. https://doi.org/10.1377/hlthaff.24.5.1214 10.1377/hlthaff.24.5.1214 16162565

[80] Branger PJ, van't Hooft A, van der Wouden JC, Moorman PW, van Bemmel JH. Shared care for diabetes: supporting communication between primary and secondary care. Int J Med Inform. 1999;53(2–3):133–42.https://doi.org/10.1016/s1386-5056(98)00154-3 10.1016/s1386-5056(98)00154-3 10193883

[81] Branger PJ, Duisterhout JS. Electronic Data Interchange in medical care: an evaluation study. Proc Annu Symp Comput Appl Med Care. 1991;58–62. PMC2247495 1807669

[82] The European Parliment. The European Health Data Space and amending Directive 2011/24/EU and Regulation (EU) 2024/2847 [Internet]. 2025. Available from:https://data.consilium.europa.eu/doc/document/PE-76-2024-INIT/en/pdf

[83] U.S. Department of Health and Human Services, Office of the National Coordinator for Health Information Technology.HealthIT.gov. 2022. The Trusted Exchange Framework (TEF): Principles for Trusted Exchange. Available from:https://www.healthit.gov/sites/default/files/page/2022-01/Trusted_Exchange_Framework_0122.pdf

[84] Solomon MR. Regional Health Information Organizations: A Vehicle for Transforming Health Care Delivery? J Med Syst. 2007;31(1):35–47.https://doi.org/10.1007/s10916-006-9041-0 10.1007/s10916-006-9041-0 17283921

[85] HL7, Boston Children's Hospital. CDS Hooks. 2018. CDS Hooks. Available from:https://cds-hooks.org/

[86] Computational Health Informatics Program, Boston Children's Hospital. SMART Health IT. 2020. SMART on FHIR. Available from:https://docs.smarthealthit.org/

[87] HL7. HL7 International. 2023. FHIR v5.0.0. Available from:https://www.hl7.org/fhir/summary.html

[88] Briscoe B, Odlyzko A, Tilly B. Metcalfe's law is wrong - communications networks increase in value as they add members-but by how much? IEEE Spectrum. 2006;43(7):34–9.https://doi.org/10.1109/MSPEC.2006.1653003

[89] Allen K, Gilliam NJ, Kharrazi H, McPheeters M, Dixon BE. Health information exchange: incorporating social and environmental determinants of health into health information exchange. In: Dixon BE, editor. Health Information Exchange [Internet]. 2nd ed. London: Academic Press; 2023. p. 423–33.https://doi.org/10.1016/B978-0-323-90802-3.00023-X

[90] Sharma NA, Chand RR, Buksh Z, Ali ABMS, Hanif A, Beheshti A. Explainable AI Frameworks: Navigating the Present Challenges and Unveiling Innovative Applications. Algorithms. 2024;17(6):227.https://doi.org/10.3390/a17060227

[91] Dwivedi R, Dave D, Naik H, Singhal S, Omer R, Patel P, *et al.* Explainable AI (XAI): Core Ideas, Techniques, and Solutions. ACM Comput Surv. 2023;55(9):194:1-194:33. https://doi.org/10.1145/3561048

[92] Pumplun L, Peters F, Gawlitza JF, Buxmann P. Bringing Machine Learning Systems into Clinical Practice: A Design Science Approach to Explainable Machine Learning-Based Clinical Decision Support Systems. J. Assoc. Inf. Syst. 2023;24(4):953–79.https://doi.org/10.17705/1jais.00820

[93] Guo Y, Guo S, Jin Z, Kaul S, Gotz D, Cao N. Survey on Visual Analysis of Event Sequence Data. IEEE T Vis Comput Gr. 2022;28(12):5091–112.https://doi.org/10.1109/TVCG.2021.3100413 10.1109/TVCG.2021.3100413 34314358

[94] Hossin MA, Kamruzzaman MM, Alrashdi I, Alsharari AM, Almjnoony A. Key Technologies, Applications, Trends of Visual Analysis and Prediction in Healthcare System. In: 2023 IEEE Globecom Workshops (GC Wkshps) [Internet]. 2023. p. 2118–22.https://doi.org/10.1109/GCWkshps58843.2023.10465101

[95] Mulligan DK, Kroll JA, Kohli N, Wong RY. This Thing Called Fairness: Disciplinary Confusion Realizing a Value in Technology. Proc ACM Hum-Comput Interact. 2019;3(CSCW):1–36.https://doi.org/10.1145/3359221

[96] Rajkomar A, Hardt M, Howell MD, Corrado G, Chin MH. Ensuring Fairness in Machine Learning to Advance Health Equity. Ann Intern Med. 2018;169(12):866–72.https://doi.org/10.7326/M18-1990 10.7326/M18-1990 PMC6594166 30508424

[97] Chen F, Wang L, Hong J, Jiang J, Zhou L. Unmasking bias in artificial intelligence: a systematic review of bias detection and mitigation strategies in electronic health record-based models. J Am Med Inform Assoc. 2024;31(5):1172–83.https://doi.org/10.1093/jamia/ocae060 10.1093/jamia/ocae060 PMC11031231 38520723

[98] Sauer LM, Resnick B, Links JL, Garibaldi BT, Rutkow L. Information Challenges Associated With Accessing and Sharing of Patient Information in Disasters: A Qualitative Analysis. Health Secur. 2023;21(6):479–88.https://doi.org/10.1089/hs.2023.0058 10.1089/hs.2023.0058 37889613

[99] Kierkegaard P, Kaushal R, Vest JR. Applications of Health Information Exchange Information to Public Health Practice. AMIA Annu Symp Proc. 2014;2014:795–804. PMC4419901 25954386

[100] Abir M, Mostashari F, Atwal P, Lurie N. Electronic Health Records Critical in the Aftermath of Disasters. Prehosp Disaster Med. 2012;27(6):620–2.https://doi.org/10.1017/S1049023X12001409 10.1017/S1049023X12001409 23009722

[101] Murdoch B. Privacy and artificial intelligence: challenges for protecting health information in a new era. BMC Med Ethics. 2021;22(1):122.https://doi.org/10.1186/s12910-021-00687-3 10.1186/s12910-021-00687-3 PMC8442400 34525993

[102] Blavin F, Ramos C, Cafarella Lallemand N, Fass J, Ozanich G, Adler-Milstein J. Analyzing the Public Benefit Attributable to Interoperable Health Information Exchange [Internet]. Washington DC: Urban Institute; 2017. Available from:https://aspe.hhs.gov/reports/analyzing-public-benefit-attributable-interoperable-health-information-exchange

