# Supplementary material for: Advancing Health Information Exchange: AI/ML, Social Needs, and Cybersecurity Trends
Source: Yearb Med Inform. 2026 Nov 3;34(1):219–31. doi: 10.1055/s-0046-1816622 (PMC13730668; doi:10.1055/s-0046-1816622)
Supplement: Supplementary file 1 [file 10-1055-s-0046-1816622-svest.pdf]

Supplemental Table A. Characteristics of literature on health information exchange, artificial intelligence, social needs, and cybersecurity trends.

Article	Topic(s)	Location	Setting / population	Design	Perspective of HIE
Ben-Assuli & Vest 2020 [43]	AI/ML	USA	Emergency department	Longitudinal cohort	Data repository
Ben-Assuli & Vest 2022 [54]	Social needs	USA	Emergency department	Longitudinal cohort	Data repository
Choi et al 2023 [68]	Cybersecurity	USA	Hospital	Longitudinal cohort	Organizational network
Dev et al 2024 [50]	AI/ML	USA	Community	Cross-sectional	Data repository
Dexter et al 2020 [42]	AI/ML	USA	Community	Cross-sectional	Data repository
Duong et al 2021 [45]	AI/ML	USA	Community	Cross-sectional	Data repository
Esmaeilzadeh 2022 [73]	Cybersecurity	USA	Providers	Qualitative	Process
Gingold et al 2021 [55]	Social needs	USA	Hospital	Longitudinal cohort	Data repository
Guo et al 2023 [56]	Social needs	USA	Emergency department	Longitudinal cohort	Data repository
Holler et al 2023 [46]	AI/ML	USA	Community	Cross-sectional	Data repository
Holt et al 2023 [65]	Social needs	USA	Screening tools	Case study	Data repository
Ido et al 2022 [67]	Cybersecurity	Japan	Hemodialysis facilities	Case study	Process & Organizational network
Kasthurirathne et al 2019 [47]	AI/ML	USA	Primary care	Cross-sectional	Data repository
Kasturi et al 2021 [49]	AI/ML	USA	Community	Cross-sectional	Data repository
Khurshid et al 2023 [62]	Social needs	USA	Community	Case study	Process & Organizational network
Korzeniewski et al 2020 [63]	Social needs	USA	Community	Technical description	Process & Organizational network
Lee et al 2022 [74]	Cybersecurity	Korea	Providers, patients, and developers	Qualitative	Process
Maguire et al 2024 [57]	Social needs	USA	Hospital	Cross-sectional	Organizational network
Mamlin et al 2021 [66]	Cybersecurity	USA	Disaster clinics	Case study	Process
Mazurenko et al 2024 [58]	Social needs	USA	Emergency department	Cross-sectional	Data repository
McMoran et al 2022 [51]	AI/ML	USA	Providers & patients	Cross-sectional	Organizational network
Mezher et al 2021 [72]	Cybersecurity	International	Community	Case study	Process
Rana et al 2024 [59]	Social needs	USA	Community	Cross-sectional	Data repository
Richwine et al 2024 [61]	Social needs	USA	Hospital	Cross-sectional	Data repository
Sandoval et al 2024 [60]	Social needs	USA	Hospital	Cross-sectional	Data repository
Shen et al 2019 [75]	Cybersecurity	Canada	Patients	Qualitative	Process
Su et al 2021 [52]	AI/ML	USA	Home health	Technical description	Process
Vázquez et al 2024 [48]	AI/ML	Spain	Community	Cross-sectional	Data repository
Vest & Ben-Assuli 2019 [44]	AI/ML & Social needs	USA	Emergency department	Cross-sectional	Data repository

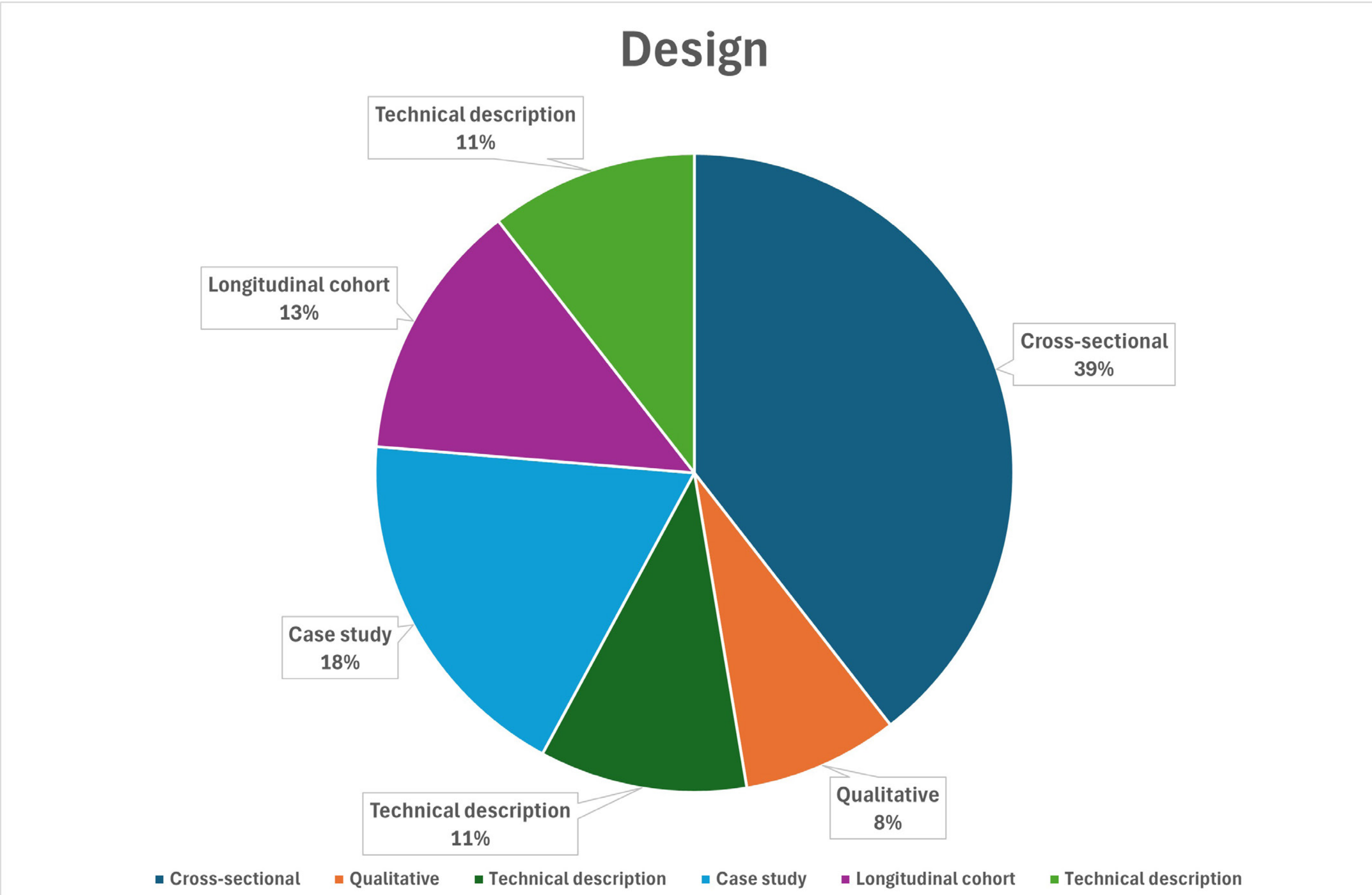

Supplemental Figure A. Articles by study design.

Vest et al 2022 [64]	Social needs	USA	Researchers	Cross-sectional	Data repository
Zahoor et al 2024 [53]	AI/ML	Pakistan	Outpatient	Technical description	Process
Zhuang & Zhang 2023 [71]	Cybersecurity	USA	Community	Technical description	Process
Zhuang et al 2020 [70]	Cybersecurity	USA	Community	Case study	Process
Zhuang et al 2023 [69]	Cybersecurity	USA	Community	Case study	Process
